# AI-Driven Design and Comparative Evaluation of SNEDDS for the Optimized Nanoencapsulation of Phytoextracts

**DOI:** 10.3390/nano16130793

**Published:** 2026-06-26

**Authors:** Cassandra G. Prieto-Medrano, Gildardo Sanchez-Ante, Araceli Zavala, Angélica Lizeth Sánchez-López, Adriana Cavazos-Garduño, Ana Karina Carrillo-Pérez, Rebeca Garcia-Varela, Yocanxóchitl Perfecto-Avalos

**Affiliations:** 1 School of Engineering and Sciences, Tecnologico de Monterrey, Av. General Ramon Corona 2514, Nuevo Mexico, Zapopan CP 45138, Jalisco, Mexico; cassandra.prieto.medrano@exatec.tec.mx (C.G.P.-M.); gildardo.sanchez@tec.mx (G.S.-A.); araceli.zavala@tec.mx (A.Z.); alsl@tec.mx (A.L.S.-L.); a01636075@tec.mx (A.K.C.-P.); 2 Centro Universitario de Ciencias Exactas e Ingenierías, Universidad de Guadalajara, Guadalajara CP 44430, Jalisco, Mexico; adriana.cavazos@academicos.udg.mx; 3 Department of Cell & Regenerative Biology, Carbone Cancer Center, School of Medicine and Public Health, University of Wisconsin-Madison, Madison, WI 53792, USA

**Keywords:** self-nanoemulsifying drug delivery systems, machine learning, anacardic acids

## Abstract

Oil-in-water nanoemulsions (NE) can increase the water solubility of plant-derived bioactive molecules as drug candidates. Machine learning-guided NE design can prevent the expensive, time-consuming trial-and-error process. NE composition data was aggregated into a dataset; a predictive machine learning model identified improved self-nanoemulsifying system formulations (olive oil and combinations of Tween 20, Tween 80, glycerol, and soy lecithin). Predictive power was assessed by estimating successful self-nanoemulsification through transmittance and Dynamic Light Scattering. NEs were loaded with an organic extract containing anacardic acid. Encapsulation efficiency was measured by UHPLC. Antiproliferative activity was evaluated on human hepatic cancer (Hep G2) and normal-like human embryonic kidney (HEK-293) cell lines. The model showed an accuracy of 81%. The best-performing formulation, consisting of 10% olive oil, 60% Tween 20, and 30% glycerol, exhibited an average particle size of 162.8 ± 26 nm, a polydispersity index of 0.234 ± 0.03, and high encapsulation efficiency. While HEK-293 cells remained unaffected, naked NE exhibited a selective growth inhibitory effect on the Hep G2 cell line. Loaded NE increased the cytotoxic effect on Hep G2 (IC50: 5.9 ± 1.27 µM). Machine learning-guided NE formulation was a successful carrier for the plant extract and the molecule of interest, providing a proof of concept for how artificial intelligence can shorten the development pipeline for NE drug delivery systems.

## 1. Introduction

Even though current technology enables the development of new synthetic molecular entities for therapeutic use, interest in natural sources of potential therapeutic agents has not waned. Over 1800 new drugs have been approved over the last two decades, with 50% being of natural origin, derived from plants or microorganisms [1]. The drug discovery process can be guided to some extent by traditional medicinal botany, especially in countries with both great biodiversity and extensive use of herbal medicines, such as Mexico. However, the usefulness of this preexisting traditional knowledge is often limited by a lack of scientific evidence, and even when in vitro activity is experimentally supported, it often does not translate to in vivo activity [2]. Although there are many reasons why a potential therapeutic agent may have low in vivo activity despite showing good in vitro activity, two problems commonly seen in plant bioactive molecules are low water solubility and high molecular weight, which contribute to low absorption and poor bioavailability [3,4].

Such is the case of anacardic acids, a family of molecules extracted from many plant sources, including the bark of *Amphipterygium adstringens.* In traditional Mexican medicine, the pulverized bark of *A. adstringens* is used to treat a variety of diseases and conditions, including superficial wounds, gastritis, gastric ulcers, colic, fever, tooth pain, and gastrointestinal cancer [4,5,6]. Specifically, anacardic 6-pentadecyl salicylic acid (6SA) has attracted researchers’ attention for its activity as both an antiproliferative and antineoplastic agent. Research has shown that co-administration of 6SA with first-line chemotherapeutic agents increases the antitumoral activity against breast cancer, while decreasing the chemotherapy-induced blood cell toxicity; this results in improved survival in animal models treated with Taxol [7], 5-fluorouracil, and carboplatin [5]. Due to the presence of long hydrocarbon chains, anacardic acids are strongly lipophilic compounds, with estimates placing their logarithmic water-octanol partition coefficient (logP) at around 9.9 [8]. As a result, aqueous and alcoholic extracts of *A. adstringens* bark generally exhibit poor bioactivity even in vitro [9]. Although in vivo assays in murine models have demonstrated the therapeutic potential of hexane-extracted anacardic acids in drug-resistant breast cancer, both as monotherapy and adjuvant in Taxol treatment [5,7], none have yet been approved for clinical trial, as a result of pharmacokinetic concerns surrounding the highly lipophilic character, binding to plasmatic proteins, possible hepatotoxicity [10], and acidic pH [11]. Nonetheless, pharmacokinetic analysis suggests potential therapeutic applications of anacardic acids [12] and attempts at improving the pharmacokinetics of anacardic acids have generally been directed towards synthesizing derivatives with slightly altered physicochemical properties [10]. This approach, however, introduces significantly more candidate molecules that require individual characterization, increasing the time and resources expended.

Nanoscale drug delivery systems have been proposed as a means to overcome the poor pharmacokinetics of various drugs. In the specific case of poor bioavailability, due to low water solubility, oil-in-water nanoemulsions (NEs), here the drug is encapsulated in lipid globules stabilized within an aqueous medium by surfactants, have become an interesting alternative due to their high biocompatibility, innate ability to solubilize hydrophobic drugs, and increased penetration into highly resistant tissues [13,14]. The aforementioned characteristics provide key technical advantages to the finished pharmaceutical product, such as a reduction in adverse effects, higher response at lower apparent administered dosage, improved localized delivery, and ease of modification into smart nanomaterials, allowing for more personalized treatment options [15].

The potential of NEs as drug delivery systems is undermined by challenges in scaling up production processes. Broadly speaking, current manufacturing approaches focus either on producing an emulsion with a larger particle size that is then reduced mechanically, or on selecting an oil-to-surfactant-to-water ratio that results in the spontaneous formation of an NE. In the first approach, referred to as high-energy, the cost of machinery and energy required to reduce particle size limits the economic feasibility of the large-scale process. The second approach, known as low-energy, typically yields NEs that are highly surfactant-rich and low in oil, raising regulatory concerns due to potential irritation and reducing drug-loading capacity [15]. While further research into low-energy, low-surfactant manufacturing methods is necessary to exploit the advantages of NE drug delivery systems fully, this is complicated because research in the field relies on trial-and-error experiments, in which dozens of composition ratios must be tested to determine the composition ranges within which a stable NE can exist. Each potential formulation requires characterization.

Recently, the Quality-by-Design (QbD) approach has gained popularity in the development of NE drug delivery systems. In this approach, the parameters that guarantee the final product meets critical characteristics are identified during the design phase and monitored throughout development.

A natural extension of current trends in NE development is the use of machine learning (ML) technologies to guide formulation design. Application of ML to the development of nanoparticle drug delivery systems is recent, and gaining favor as they allow for predictions of key parameters such as drug loading capacity, particle size, release kinetics, stability, and surface charge, based only on the physicochemical properties of the system components [16]; reducing the time and cost of production while ensuring quality [17]. Although ML has been used to generate other lipid-based nanoparticle drug delivery systems, information in the literature is still sparse.

This work aims to streamline the design of self-nanoemulsifying drug delivery systems for bioactive molecules extracted from plants using ML. Various algorithms were trained and tested on a database compiled from independent published studies that used a common ultrasonication method to mix oil-phase components. A series of formulations predicted by the best algorithm was tested experimentally to assess the model’s accuracy. Concurrently, hexane extracts from *A. adstringens* were prepared and analyzed for anacardic acid (AA) content to serve as bioactive cargo. Following initial testing, a successful SNEDDS formulation was selected based on solution transmittance as an indicator of particle size, quantitative particle-size data, polydispersity index measured by dynamic light scattering, and extract-loading capacity. Finally, to demonstrate the feasibility of this optimized delivery system, the antiproliferative activity of the loaded SNEDDS was evaluated in a human carcinoma cell line and a non-transformed cell line, establishing a workflow from prediction to biological validation.

## 2. Materials and Methods

### 2.1. Reagents and Biological Samples

*A. adstringens* bark sample was purchased from Pacalli (Monterrey, Nuevo León, Mexico). Tween 20 and Tween 80 were kindly sent as samples by Croda (Snaith, UK). Cosmetic-grade olive oil was purchased from Gardenia Naturals (Guadalajara, Jalisco, Mexico). Hexane from Karal (Leon, Guanajuato, Mexico), HPLC-grade acetonitrile from Burdick & Jackson (Muskegon, MI, USA), HPLC-grade acetic acid from Tedia (Fairfield, OH, USA), and anacardic acid (95% purity) were sourced from Santa Cruz Biotechnology (Dallas, TX, USA). HEK-293T (ATCC CRL-3216) and Hep G2 cells (ATCC HB-8065) cell lines were used. Dulbecco’s Modified Eagle Medium (DMEM), penicillin-streptomycin 100X solution, phosphate-buffered saline, fetal bovine serum, dimethyl sulfoxide (DMSO), glycerol, and trypsin-EDTA at 0.05% (1X) with phenol red were sourced from Sigma-Aldrich (Saint Louis, MO, USA). An MTS assay kit (CellTiter 96) was purchased from Promega (Madison, WI, USA).

### 2.2. Data Acquisition

Following the methodology proposed by Amasya and colleagues [18], the Critical Quality Attributes (CQAs) for the NE formulations were defined as a mean particle size (MPS) of 200 nm or less and a polydispersity index (PDI) of 0.3 or less. To provide examples, data were manually extracted from a curated selection of literature [19,20,21,22,23,24,25,26,27,28,29,30,31,32,33,34,35,36,37,38,39,40,41,42,43,44,45,46] which met the following criteria:

- Published in a peer-reviewed journal indexed in Web of Science or Scopus;
- Described methodology in sufficient detail to ascertain that the formulation prepared can be classified as an SNEDDS;
- Contained sufficient characterization of the SNEDDS, including at the very least mean particle size and PDI;
- The composition of oil, surfactant, and cosurfactant was provided in percentage by weight, or sufficient information on the preparation was provided that the composition units could be converted to percentage by weight.

Formulations were labeled successful (1) if they met the CQAs, and unsuccessful (0) otherwise, to yield a dataset with balanced classes. Composition values (the amount of each component added to the NE mixture), reported in the literature as weight percent, were left as is. To describe the identity of the surfactant components in the system, hydrophilic-lipophilic balance (HLB) was chosen; total polar surface area (TPSA) was used as a feature to describe the oil and the surfactants. The result was a dataset with eight continuous numerical input features and 496 entries (available upon request via email to the corresponding author).

### 2.3. Model Development

The model was developed as a binary classification task by assigning a flag value of 1 to all successful systems and 0 to all unsuccessful systems. Following the analysis, one feature was deemed redundant and removed from the dataset. The clean, formatted dataset was loaded into a Jupyter notebook and split using the stratified K-fold split function in the Scikit library [47].

A total of 7 architectures were used: logistic regression, decision trees, support vector machine (SVM), random forest classifier, histogram gradient boosting classifier, extremely randomized trees classifier, and a SVM bagging classifier. The accuracy, precision, recall, and F1 score were calculated for each algorithm’s predictions on the test set. Additionally, the difference in accuracy between the test set and training set was calculated. A confusion matrix and a receiver operating characteristic (ROC) curve were generated for each algorithm.

### 2.4. Model Implementation

The selected components were cosmetic-grade olive oil and surfactants, including soy lecithin, glycerol, Tween 80, and Tween 20. A dataset comprising 15 combinations of olive oil, a surfactant, and a cosurfactant, each with ingredient concentrations ranging from 5% to 90%, was synthetically generated in Python 3.13.3. Following input creation, data was loaded into the Jupyter notebook containing the trained model using the classifier architectures listed above. After flag value prediction, the model’s outputs were used to create ternary plots using the Plotly library [48].

### 2.5. Validation of Model Output

To validate the results of the ML model, three points per system were prepared. The points were selected using an Extreme Vertices Mixture designed [49] in Minitab (v21.4, Minitab LLC, 2023), limiting the composition by weight to a minimum of 30% and a maximum of 60% for both surfactant and cosurfactant, and a minimum of 10% and a maximum of 40% for the oil. The bounding values for the oil percentage were chosen based on the average and maximum values reported in the literature for oil content [50], while the surfactant content boundaries were chosen to complement said oil content values. This design was considered appropriate in this context because the compositions of the NE formulation were expressed as percentages by weight; the Extreme Vertices Mixture design allows exploration of mixture behavior while bounding the possible combinations of compositions so that the sum of all three is still 100%. The same points generated were tested in triplicate for each system, for a total of 54 experimental runs. The six different systems were evaluated as blocks in the experimental design.

### 2.6. Extract Preparation

A mixture of anacardic acids was extracted from ground *A. adstringens* bark with hexane by Soxhlet extraction for 12 cycles, then excess solvent was removed using a rotary evaporator. Extractions were partitioned into two groups: a single pool (labeled P) consisting of three combined batches, and three individual batches (labeled B1, B2, and B3). All samples were stored in the dark at 4 °C until use.

### 2.7. UHPLC Analysis

Samples were analyzed using reverse-phase liquid chromatography on ACQUITY UPLC H-Class equipment (Waters, USA) as per Oiram et al. [51]. Analysis was performed under isocratic flow at 0.8 mL/min for 15 min, with 80% acetonitrile and 20% water as mobile phase. Both components were acidified with 1% glacial acetic acid. The samples were kept at 4 °C, and 5 µL of each sample was injected at a time. Samples were separated on a C18 column (Cortecs, 4.6 × 50 mm, 2.7 µm, Waters, Milford, MA, USA), which was maintained at 40 °C during injection. Detection was carried out using a Photodiode Array detector at 243 nm, the highest peak in the absorption spectrum reported for anacardic acid [51]. The calibration curve was prepared at 5, 20, 40, and 100 µg/mL.

### 2.8. Nanoemulsion Preparation and Characterization

Three replicates were prepared per SNEDD formulation from the validation section using the method described by Bravo-Alfaro et al. [52]. Briefly, anhydrous NE components were added to a final mixture mass of 100 mg, then ultrasonicated for 5 min in an ultrasound water bath, followed by the addition of 10 mL of water and another 5 min of ultrasonication.

The resulting NE were evaluated using a UV-Vis spectrophotometer (SP-UV1100, DLAB, Kuala Lumpur, Malaysia) for transmittance at 470 nm—one of the absorption peaks for the olive oil in the formulation—and evaluated for mean particle size and polydispersity index [53] using Dynamic Light Scattering (DLS, Zetasizer Nano SZ, Malvern, UK). For the DLS analysis, 200 µL of each sample was diluted in 1800 µL of distilled water and analyzed at 25 °C at a 90° angle for 17 runs per replicate. The measurements were carried out in triplicate.

For the loaded NE, the total amount of extract to be added into the anhydrous mixture was determined spectrophotometrically. The NE from the model validation section with the highest transmittance percentage was reproduced and loaded with 10, 20, 30, 50, and 100 µL of extract. The maximum loading capacity of the system was considered to be the highest amount of extract added at which the resulting NE’s transmittance remained unchanged with respect to the unloaded formulation.

Encapsulation efficiency at the highest carrying capacity was determined by NE centrifugation at 4000 RPM for 10 min, followed by UV-Vis quantification of anacardic acid in the supernatant at 310 nm [54] using first a spectrophotometer, and later UHPLC as described in Section 2.7.

### 2.9. Cytotoxicity Assays

Hep G2 and HEK-293T cells were cultured in low-glucose DMEM with 10% fetal bovine serum and 1% penicillin-streptomycin in a humidified atmosphere at 37 °C with 5% added CO_2_. Cells were passaged when confluence reached 80% and 90%.

Cytotoxic activity for the unloaded NE, the loaded NE, the extract, and pure anacardic acid on Hep G2 and HEK-293T was evaluated using the MTS method [55]. DMSO was used as a positive control. To verify that the observed cytotoxicity was due to the treatment and not starvation associated with reduced culture media volume, a PBS treatment was used as a control. Cells were seeded at 10,000 cells per well and incubated for 24 h in a humidified atmosphere at 37 °C with 5% CO_2_. Following this period, the culture media was removed and replaced by 35 µL of the treatments at 1, 10, 20, and 50 µM, calculated based on anacardic acid content or DMSO; each well was then filled to 100 µL by adding 65 µL of complete media, and the plates were incubated once again for 24 h. After the second incubation period, 20 µL of MTS reagent was added per well, and the plates were incubated for 3 h before being analyzed in a plate reader at 490 nm. All assays were conducted in triplicate. Cell viability curve was fitted to a four-parameter logistic growth equation, shown below as Equation (1), using the Matlab Curve Fitting Toolbox 3.9 (R2023a, MathWorks):

(1) $$\%\;Cell\;viability=\frac{b+(b-a)}{1+e^{\frac{x+c}{d}}}$$

where *a*, *b*, *c*, and *d* are parameters calculated by the curve fitting process, and *x* is the dose expressed in µM. The half maximal inhibitory concentration (IC50), that is, the concentration of a treatment needed to reduce the cell viability by half, was calculated by solving the equation for *x* and substituting the parameters and corresponding cell viability into it.

### 2.10. Statistical Analysis

Statistical analysis was performed using Minitab. A one-way analysis of variance (ANOVA) using Welch’s test at 95% significance level was applied to the IC50 values to determine whether the treatments caused statistically different responses in both cell lines. The treatments were grouped for comparison using the Games–Howell method.

## 3. Results

### 3.1. Baseline Model Analysis 

After evaluating the performance of all seven ML algorithms, it was found that there was no significant difference in computational efficiency, as measured by training time. There were, however, significant differences in accuracy, precision, recall, F1 score, and difference between the test and training datasets, as shown in Table 1.

**Figure 1:**
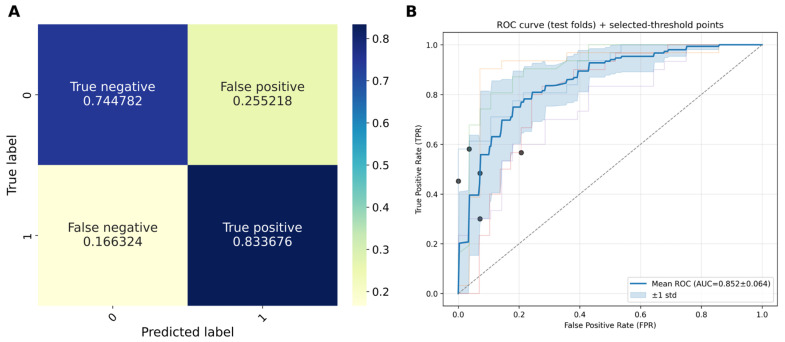
Performance metrics for the bagging SVM model. (**A**) Confusion matrix with the mean proportions of True Negatives (TN), False Positives (FP), False Negatives (FN), and True Positives (TP) obtained across the outer test folds. (**B**) Receiver Operating Characteristic (ROC) curves obtained on the outer test folds of the nested cross-validation. Thin colored lines correspond to the ROC curve of each individual test fold, while the thick blue line represents the mean ROC across folds; the shaded band indicates ±1 standard deviation of the true positive rate (TPR) at each false positive rate (FPR). The diagonal dashed line denotes chance-level performance. Black markers indicate the selected operating points (one per fold) corresponding to the decision thresholds tuned to control FPR during the inner-loop validation and then evaluated on the held-out test folds. Overall discrimination is high, with a mean ROC-AUC of 0.852 ± 0.064 across folds.

A closer look at other performance metrics for the bagging SVM model is provided in Figure 1, showing the confusion matrix and ROC curve for the training dataset validation subsets. ROC curves and confusion matrices of the remaining six models are shown in Supplementary Figures S1 and S2.

Both the gradient boosting algorithm and the support vector machine bagging algorithm showed scores higher than 80% across the board for the metrics shown in Table 1; the choice of one over the other was made given the difference between the accuracy achieved with the test data subset and the training dataset, with the value seen for the bagging SVM being much lower and; therefore, suggesting better generalization of the model to datapoints outside the training dataset [56]. This is due to the projected use of the model for predicting de novo formulations. SVM in nanostructure production with pharmaceutical applications: additionally, working effectively with the prediction of particle size and zeta potential based on the compound proportion, has also shown vast application and predictive capacity for the classification of drug-carrier compatibility [57]. This kernel-based method focuses on the emulsion properties, making it an efficient tool for the optimization of the formulation process [17], enhancing the predictive capacity by bagging.

The bagging SVM performed well on Area Under the Curve (AUC), with a mean value across all folds of 0.87. Examination of the confusion matrix for the bagging SVM model shows that at the selected threshold, the observed true negative rate was 0.74 and the true positive rate was 0.83. To determine the operating threshold, we selected, within each outer cross-validation fold, a threshold on the internal validation split to control the false positive rate (FPR) at ≤X (X = 0.10 in this case). We then applied this threshold to the corresponding held-out test fold and reported the resulting operating point on the ROC and precision–recall curves computed from that test data. Table 2 shows the corresponding metrics for each fold. When no threshold met the FPR constraint in the internal validation, the threshold that minimized the FPR (and, in the event of a tie, maximized recall) was selected, as in Fold 4.

Under nested cross-validation with a threshold selection aimed at controlling the false positive rate (FPR), the model achieved an average performance (mean ± standard deviation, across the external folds) of ROC-AUC = 0.852 ± 0.064 and AP (PR) = 0.838 ± 0.077. By setting the decision threshold according to this criterion, we obtained accuracy = 0.690 ± 0.052, precision = 0.877 ± 0.092, recall = 0.477 ± 0.101, and F1 = 0.610 ± 0.092.

**Figure 2:**
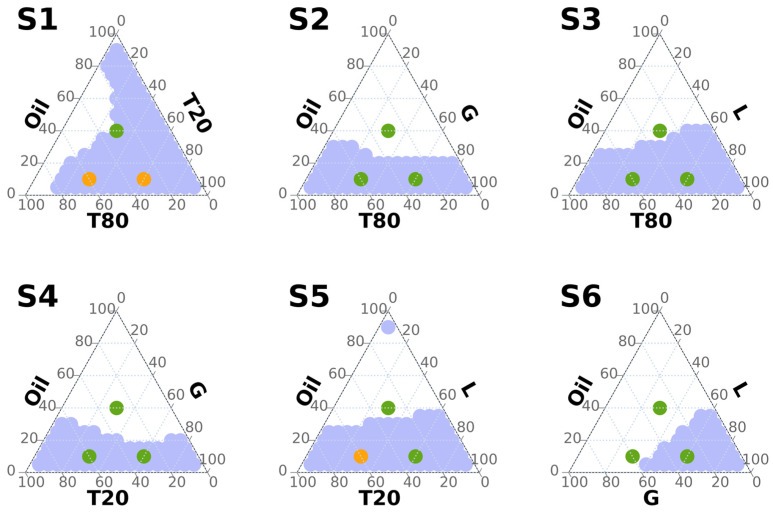
Ternary phase diagrams mapping the self-nanoemulsifying regions. Purple–gray markers represent formulations that the model predicted to fall within the self-nanoemulsifying region. Green markers indicate formulations in which DLS confirmed successful nanoemulsion formation. Orange markers represent formulations in which the experimental data did not match the model’s prediction; in other words, although the model placed them within the nanoemulsion zone, DLS showed they failed to form a stable nanoemulsion. T80 means Tween 80, T20 is Tween 20, G is glycerol, and L is lecithin.

These results suggest that by prioritizing control of false positives through threshold adjustment, the model maintains high precision at the expense of moderate sensitivity, consistent with the objective of operating under a conservative positive-prediction regime. Figure 2 shows the ROC curve with the selected thresholds for the test folds. The model appears to perform slightly better at correctly predicting when a formulation will be successful. The context of the application can help determine whether it is riskier to have more false positives than false negatives [58]; here, there is a higher risk of a false positive, given the potential waste of resources in preparing a formulation that will not satisfy the CQAs. However, the overall model performance remains significantly better than random chance in all cases, representing an improvement over the naïve formulation approach.

### 3.2. Experimental Validation of the Machine Learning Model

To assess whether the model output agrees with experimental data, the bagging SVM model was used to predict outcomes for the synthetic formulation dataset described in Section 2.4. The combinations of oil, surfactant, and cosurfactant were grouped into six systems, as shown in Table 3.

Of the entire design space explored for each system, three formulations per system were selected according to the Extreme Vertices Mixture Design. The boundaries of this design outline a region of the pseudo-ternary phase diagram in which, based on literature results, at least one point should be within the nanoemulsification region.

Analysis of the selected formulations using DLS to measure MPS and PDI allowed the estimation of the model’s experimental accuracy. Figure 2 shows that the bagging SVM model had an overall experimental prediction accuracy of 83%. Each system presents the boundaries of this design. For example, the purple–gray markers across all systems represent formulations that the model predicts to fall within the self-nanoemulsifying region, while the green markers symbolize the confirmation of the DLS model’s prediction. However, the prediction failures (orange markers) are most likely due to limitations in the model’s training approach, as there are no reports in the literature that combine Tween 20 and Tween 80. This has been observed in system 5, where the database contains only four examples of lecithin. In these cases, the model is extrapolating to systems it has not encountered to make use of the materials available for experimentation. It is worth noting that these are two cases in which both components are chemically similar to surfactants, which contradicts the theory of nanoemulsifying system design. The theory suggests choosing one surfactant with a high HLB and another with a low HLB so that one preferentially partitions into the lipid phase and the other into the aqueous phase, thereby stabilizing the interface. Significantly, systems that failed in experimental validation had higher Tween 20 content, whereas systems combining Tween 80 and lecithin showed no prediction errors.

### 3.3. UHPLC Analysis of the Hexane Extract of A. adstringens

**Figure 3:**
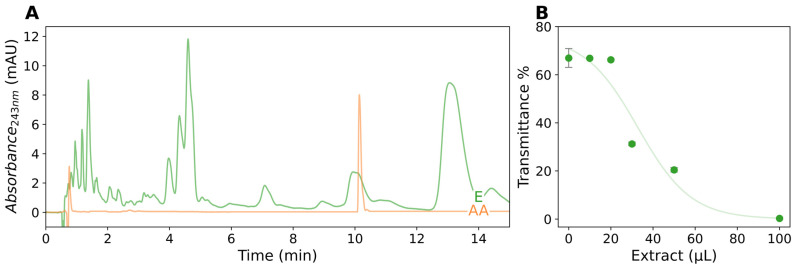
Extraction and loading of anacardic acid. (**A**) Reference chromatogram of pure anacardic acid (AA) and chromatograms for the three batches of extract (E) from *A. adstringens*. (**B**) Transmittance as a function of the amount of *A. adstringens* extract in the oleous phase. AA anacardic acid, E extract.

Figure 3A compares the chromatogram of the pure anacardic acid standard with the samples analyzed. The reference chromatogram shows a single peak with a retention time of 10.065 min. The sample chromatograms, by contrast, showed a peak with a retention time of around 9.9 min for all three batches and the pool, as shown in Table 4. Considering this is a crude, unpurified extract, the presence of many other unidentified peaks is expected. Nevertheless, in accordance with the published literature, the peak observed around 13 min may correspond to the presence of anacardic acid molecules with different numbers of unsaturated carbon bonds in the side chain; the peak that shares the standard retention time is attributed to the triene, and the peak at 13 min could be due to the diene [51,59].

Although the observed retention time of the extracted samples was slightly lower than that of the pure standard solution, the difference was only 1.6%. In the published article, which served as the basis for the UHPLC method used here, the authors showed chromatograms where the retention time of the analyzed sample changed by 1.3% compared to the retention time of the pure standard, yet these values were considered sufficiently similar to be associated with the same molecule as per the criteria of the International Conference on Harmonization [51]. Consequently, this difference is not significant in this analysis, and both peaks can reasonably be attributed to the same molecule.

For the pool sample, 90 g of ground bark from *A. adstringens* was used. Each of the following batches was obtained from 15 g of bark. Following concentration in a rotary evaporator, the pool sample yielded 1.1 g of a thick green paste. From this pool sample, UHPLC analysis yielded an anacardic acid content of 19.350 µg, equivalent to 0.0002% of the total bark weight. According to reports, *A. adstringens* bark contains 0.96 g of anacardic acid per kilogram, or 0.0009% by weight [5]. It is important to note that, as with all extracts obtained from biological material, circumstantial factors may significantly affect the amount of a molecule of interest available for extraction; in the case of *A. adstringens*, the age of the tree, the sex of the plant, and the humidity around the time of collection are known to affect the chemical profile [60]. Additional factors affecting the extraction yield include the use of hot solvent: reportedly, up to 33% of anacardic acid is lost upon heating at 140 °C [61].

### 3.4. Nanoemulsion Preparation and Loading

Based on the results obtained in the initial transmittance analysis and DLS measurements (Table 5), one system was selected from the formulations for further testing. This formulation, referred to going forward as NE, was composed of 10% olive oil, 60% Tween 20, and 30% glycerol. For comparison, the highest percentage of oil content registered as a successful nanoemulsion formulation in the dataset used to train the ML models was 70%, achieved by Xue et al. following an optimization of a formulation containing isopropyl myristate as oil, Tween 80 as surfactant, and 1,2-propanediol as cosurfactant [45]. The highest observed oil content registered using olive oil was 27%, achieved by Imada et al., following a naïve approach based on iterative exploration of a pseudo-ternary phase diagram [62]. Consequently, while the physicochemical properties of the selected NE are in agreement with the established CQAs, it is entirely possible and indeed likely that a more thorough examination of the original model predictions may have yielded a formulation with a higher oil content, with the benefits of a lower surfactant content (relevant for potential clinical applications) and higher loading capacity.

To determine the maximum loading capacity of the NE, the transmittance method was used as an initial indicator before DLS characterization. Figure 3B shows a plot of the transmittance of the prepared NE versus the amount of the extract present in the oily phase. The transmittance remained unchanged relative to the naked NE up to a total of 20 µL of the extract pool, then rapidly decreased after 30 µL. Given the concentration of anacardic acid in the pool sample (Table 5), the total carrying capacity of the NE was calculated as 0.048 mg of anacardic acid per mL of NE, or 138 µM. The MPS and PDI of the formulation at this concentration of extract were 153.3 ± 7.1 nm and 0.258 ± 0.061, respectively. This suggests that the amount of extract added to the NE does not significantly alter the properties observed in the naked NE.

The initial UV-Vis analysis of encapsulation efficiency for the NE at the maximum loading capacity was inconclusive, as the absorbance limit of 4-digit values suggested that there was no anacardic acid in the aqueous phase after centrifugation. Further UHPLC analysis detected no anacardic acid in the aqueous phase. Using the methods employed in this work, the selected formulation appears to achieve high encapsulation efficiency. The method used to separate the phases, or the time used, may have been a determining factor for this result: further analysis using dialysis methods, for example, may yield data to contradict this [20].

### 3.5. Cytotoxicity Assay

**Figure 4:**
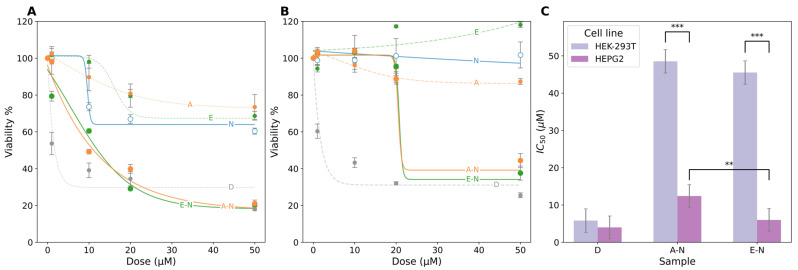
Cell viability as a function of treatment dose: (**A**) HEK-293T cell line; (**B**) Hep G2 cell line. (**C**) Comparison of means between the two cell lines for each treatment. ** indicates *p* < 0.01, *** indicates *p* < 0.0001. E extract, E-N extract loaded NE, AA anacardic acid, A-N anacardic acid loaded NE, N naked NE, D DMSO.

For both the normal-like HEK-293T and the cancerous Hep G2 (Figure 4A,B, respectively), only three of the applied treatments caused the cultures to reach 50% viability or less: the DMSO control, the NE loaded with extract, and the NE loaded with pure anacardic acid. Since experimental results showing at least 50% viability are required to calculate a meaningful IC50 value, values are reported only for these three treatments. Nonetheless, four-parameter logistic growth models were fit to all treatments for visualization purposes.

Interestingly, in Figure 4A, the *A. adstringens* extract showed a proliferative effect not observed with pure anacardic acid. This suggests that other components in the chemical profile of the hexane extract of the bark may exert a proliferative effect on the normal-like cell line, which is not observed in the cancer cell line. Published literature reports that some of the components in the non-polar extracts from *A. adstringens* bark, such as 3α-hydroxymasticadienoic acid, improve wound closure by promoting proliferative processes [9,63]; however, it is unclear whether this particular effect can be attributed to this compound given the data collected here.

The IC50 values calculated using the four-parameter logistic growth model, along with the model fit R^2^, are shown in Table 6 and Table 7. Overall goodness-of-fit was slightly better for the Hep G2 observations; for both cell lines, the DMSO treatment exhibited a decreased goodness-of-fit, with a value below 0.9. As expected from the plots shown above, the IC50 values observed in the HEK293T cell line are larger than those seen in the Hep G2 cell line (*p* < 0.001) (Figure 4C), meaning that the Hep G2 line is more sensitive to all treatments except the DMSO control. The difference is particularly notable in the NE loaded with the extract. No statistically significant difference was found between the extract-loaded NE and anacardic acid-loaded NE for the normal-like cell line (*p* > 0.05); this is not the case with the cancer cell line.

## 4. Discussion

This study demonstrates the feasibility of an ML-guided workflow to optimize the formulation of self-nanoemulsifying systems from heterogeneous data derived from literature. As previously reported, ensemble machine learning methods generally performed better than individual methods [64,65]. For instance, in their work on developing a decision support system for microemulsion formulation, Mendyk et al. achieved an accuracy of 85% with an Artificial Neural Network and 86% with a Random Forest algorithm, both of which used a database consisting of records reported in the literature and needed 17 input variables [66]. While previous data are not focused on the same type of system modeled here, the algorithm developed in this work achieved comparable prediction performance with considerably lower computational complexity. Although bagging raises the proposed models’ complexity, it improves the predictive accuracy and ensures computational time and cost efficiency [67].

To clarify the interpretation of our results, we distinguish between (a) model discrimination and (b) operating-point selection. Discrimination reflects the classifier’s intrinsic ranking ability (e.g., ROC-AUC), whereas operating-point selection determines how decision outcomes are derived from the continuous decision scores. Consequently, threshold adjustment does not alter the underlying ranking performance, but it can substantially improve decision-level utility by explicitly trading off false positives versus false negatives according to the experimental cost structure. In our setting, false positives are particularly costly because they trigger unnecessary formulation preparation and characterization; therefore, threshold tuning improves pipeline resource efficiency by reducing non-productive experiments while maintaining sensitivity at an acceptable level. Importantly, threshold tuning is computationally negligible (a post-processing step) and does not affect training time. In addition, complementary approaches can improve predictive accuracy while preserving computational efficiency: (i) probability calibration to obtain better-calibrated probabilities and thus more reliable thresholding; (ii) cost-sensitive learning, e.g., a class-weighted SVM, to directly penalize false positives during optimization; (iii) nested cross-validation to select thresholds (and calibration/hyperparameters) robustly and avoid optimistic bias; and (iv) feature refinement and data curation/augmentation to reduce label noise and improve generalization.

ML-assisted optimization of drug delivery systems is a growing area of research. In NE, the Artificial Neural Network is the most widely used method [65], with studies focusing on stability, cytotoxicity, process parameters, ingredient concentrations, and characteristics [68,69,70]. Supervised learning ML algorithms have also shown promising predictive power for optimizing other drug delivery systems [71,72]. In this study, a bagging SVM model was selected due to its robustness and predictive capacity after a comparative evaluation against other candidate models. SVM has been increasingly used on small datasets, such as the one in the present study, making it a promising approach for NE process optimization [65].

The resulting NE formulation predicted by the developed ML model, in addition to having desirable proportions of oil and surfactant, avoids potential toxicity in clinical settings and achieves a high drug-loading capacity [15]. While existing models usually characterize unloaded vehicles or optimize carriers for pure compounds, this workflow extends prediction to an organic extract. The observation that the predicted formulation (10% olive oil, 60% Tween 20, and 30% glycerol) maintained a low particle size and high encapsulation efficiency when loaded with *A. adstringens* demonstrates that the model remains robust when handling the chemical complexity inherent to crude natural products, resulting in a stable system that shows selective cytotoxic activity. To date, published information in the literature regarding the IC50 of anacardic acid or plant extracts containing anacardic acid for cytotoxic effects on cancerous and normal-like cell lines is scarce. It has been reported that aqueous and ethanolic plant extracts containing anacardic acids, tested for cytotoxicity in vitro against cells and human dermal fibroblasts, showed no cytotoxicity against either cell line [73]. Additionally, evidence suggests that anacardic acid inhibits cancer cell proliferation by attenuating TNF-induced NF-κB activation through its antioxidant effect: its IC50 for superoxide generation inhibition is 0.04 mM, and its IC50 for xanthine oxidase inhibition is 0.30 mM [74]. In a brine shrimp lethality test, the IC50 was found to be 347.65 µg/mL [75].

The results obtained in this study are comparable to the cytotoxicity data available for other substances with antiproliferative, antineoplastic, and anticancer activity in the same cell lines. In a study evaluating the cytotoxicity of viramidine nanoparticles against hepatocellular carcinoma, Abd-Rabou et al. determined the IC50 value of their preparation as 16.69 µM for HEP-G2 [76]. In this light, the range of IC50 values observed for the loaded NEs is consistent with those reported for established anticancer drugs in the cell line under study. While the difference observed between the cancer- and normal-like IC50 values is less pronounced in the present study, the means differ significantly at the 95% significance level, indicating a degree of selectivity in the treatments.

## 5. Conclusions

The present study shows that a SNEDDS formulation strategy integrating predictive ML was implemented and tested. Through this approach, the number of experiments needed to reach a formulation with the desired quality attributes was greatly reduced.

The selected formulation exhibited a maximum loading capacity of 138 µM, calculated based on anacardic acid, and the assays performed in this work suggest that all the anacardic acid was encapsulated into the oily phase. As a point of improvement, further analysis by other means, such as dialysis methods, would be confirmatory.

In the in vitro cytotoxicity assays, there were statistically significant differences among the naked NE, the extract, pure anacardic acid, and the loaded NE in both cell lines. Notably, the extract alone had an apparent proliferative effect on the normal-like cell line, a response not observed with pure anacardic acid treatment.

The IC50 value observed for the extract-loaded NE in the cancer cell line is comparable to values reported for anticancer drugs, suggesting that the SNEDDS approach successfully potentiated the cytotoxic activity of the extract, which was not effective on its own. Crucially, there was a statistically significant difference between the effects observed in cancer and normal-like cell lines, indicating that the developed SNEDDS conferred some degree of selectivity.

Finally, although the results of this study are promising, the full potential benefits of the formulation approach presented here remain to be seen. For example, the current implementation of the model requires an intermediate step in which users specify the desired components and their proportions to predict outcomes; in the future, the ML strategy could be refined to eliminate this input-generation step, thereby enabling a generative model to produce formulation suggestions based on the provided dataset. Naturally, a limitation of the Machine Learning strategy is its reliance on a manually curated dataset, which would require maintenance to keep up with new developments. Nonetheless, as Natural Language Processing technology improves, this limitation may not prove insurmountable. Overall, this work presents a workflow that is generalizable across many formulation contexts and can significantly reduce the burdens associated with NE development.

## Figures and Tables

**Table 1:** Evaluation of metric results for each model. The bold font highlights the optimal values in the table.

Model	Accuracy	Precision	Recall	F1	Test/Train Difference
Logistic regression	0.72 ± 0.07	0.73 ± 0.08	0.73 ± 0.08	0.73 ± 0.07	1.38%
Decision trees	0.77 ± 0.08	0.77 ± 0.08	0.76 ± 0.08	0.76 ± 0.08	2.19%
Support Vector Machine	0.79 ± 0.05	0.80 ± 0.05	0.79 ± 0.05	0.79 ± 0.05	4.47%
Random Forest	0.80 ± 0.06	0.80 ± 0.06	0.80 ± 0.06	0.79 ± 0.05	6.63%
Gradient boosting	0.80 ± 0.06	0.81 ± 0.06	0.81 ± 0.06	0.81 ± 0.06	20.83%
Extremely RT	0.80 ± 0.06	0.80 ± 0.06	0.80 ± 0.06	0.80 ± 0.06	3.36%
**Bagging SVM**	**0.81 ± 0.04**	**0.82 ± 0.04**	**0.81 ± 0.04**	**0.81 ± 0.04**	**1.54%**

**Table 2:** Performance per fold in nested CV. In each fold, the threshold was adjusted during internal validation to limit the FPR and maximize recall; metrics are reported for the corresponding test fold. ROC-AUC and AP were calculated from the model’s probabilities/scores.

Fold	Threshold	Acc	Prec	Rec	F1	ROS-AUC	AP	FPR
Fold 1	0.823	0.712	1.000	0.452	0.622	0.887	0.912	0.000
Fold 2	0.738	0.695	0.882	0.484	0.625	0.907	0.876	0.071
Fold 3	0.699	0.763	0.947	0.581	0.720	0.914	0.906	0.036
Fold 4	0.638	0.678	0.739	0.567	0.642	0.799	0.716	0.207
Fold 5	0.790	0.603	0.818	0.300	0.439	0.754	0.784	0.071

**Table 3:** Description of the components for each system used to evaluate the bagging SVM model. Olive oil was used in all cases.

System	Surfactant	Cosurfactant
S1	Tween 80	Tween 20
S2	Tween 80	Glycerol
S3	Tween 80	Lecithin
S4	Tween 20	Glycerol
S5	Tween 20	Lecithin
S6	Glycerol	Lecithin

**Table 4:** Mean, standard deviation (SD), and coefficient of variation (CV) of the retention time, area, and AA concentration from the UHPLC analysis performed on the hexane extract from *A. adstringens*.

Sample	Retention Time (min)			Area			AA Concentration (µM)		
	Mean	SD	CV%	Mean	SD	CV%	Mean	SD	CV%
Standard	9.98	0.19	2.00%	529,984	7203	1.37%	286.94	-	-
Pool	9.95	0.04	0.51%	528,867	2558	0.50%	55,523.16	0.03	0.51%
Batch 1	9.92	0.05	0.53%	51,269	633	1.23%	6376.38	0.01	1.09%
Batch 2	9.98	0.02	0.20%	47,683	504	1.06%	5982.34	0.01	0.92%
Batch 3	9.93	0.01	0.07%	54,832	497	0.91%	6767.99	0.01	0.81%

**Table 5:** Mean, standard deviation (SD), and coefficient of variation (CV) of the characterization results for the prepared NE. Formulation selected for further testing is highlighted in bold. S stands for system, O% for oil content as a percent of weight, S% for surfactant content, and C% for cosurfactant content. The bold font highlights the optimal values in the table.

S	O%	S%	C%	Transmittance (%)			Size (nm)			PDI		
				Mean	SD	CV%	Mean	SD	CV%	Mean	SD	CV%
1	40	30	30	24.11	3.10	12.87%	171.47	27.28	16%	0.280	0.024	9%
1	10	60	30	54.53	1.15	2.12%	278.30	144.05	52%	0.515	0.207	40%
1	10	30	60	5.01	0.52	10.30%	199.53	70.26	35%	0.383	0.057	15%
2	40	30	30	2.41	0.28	11.61%	163.53	8.12	5%	0.404	0.042	10%
2	10	60	30	43.35	5.25	12.10%	149.23	15.18	10%	0.285	0.013	5%
2	10	30	60	45.70	2.82	6.16%	155.93	1.07	1%	0.289	0.028	10%
3	40	30	30	2.52	0.16	6.24%	249.90	39.40	16%	0.204	0.039	19%
3	10	60	30	1.85	0.02	1.25%	189.47	5.39	3%	0.206	0.044	21%
3	10	30	60	0.79	0.08	10.27%	190.17	5.63	3%	0.225	0.028	12%
4	40	30	30	12.53	1.68	13.42%	212.77	21.44	10%	0.239	0.020	8%
**4**	**10**	**60**	**30**	**62.95**	**3.93**	**6.24%**	**162.80**	**26.46**	**16%**	**0.235**	**0.034**	**15%**
4	10	30	60	52.62	4.63	8.79%	152.93	30.74	20%	0.261	0.036	14%
5	40	30	30	10.47	0.44	4.23%	204.83	3.77	2%	0.364	0.008	2%
5	10	60	30	3.02	0.25	8.43%	205.47	7.57	4%	0.345	0.006	2%
5	10	30	60	1.68	0.18	10.58%	195.17	2.16	1%	0.262	0.025	9%
6	40	30	30	0.29	0.03	11.95%	219.07	1.65	1%	0.217	0.007	3%
6	10	60	30	1.83	0.11	5.85%	346.83	9.05	3%	0.312	0.011	4%
6	10	30	60	0.87	0.03	3.04%	182.25	11.52	6%	0.267	0.008	3%

**Table 6:** Half maximal inhibitory concentration (IC50) calculated using the four-parameter logistic growth model and associated goodness-of-fit (R^2^) for treatments applied to HEK293T cells. Reported values are the mean and standard deviation.

Treatment	IC50 (µM)	R^2^
Extract loaded NE	45.52 ± 1.46	0.9599 ± 0.0174
DMSO	1.91 ± 0.45	0.8565 ± 0.1139
AA loaded NE	48.52 ± 1.18	0.9808 ± 0.0061

**Table 7:** Half maximal inhibitory concentration (IC50) calculated using the four-parameter logistic growth model and associated goodness-of-fit (R^2^) for treatments applied to Hep G2 cells. Reported values are the mean and standard deviation.

Treatment	IC50 (µM)	R^2^
Extract loaded NE	5.99 ± 1.27	0.9971 ± 0.0026
DMSO	1.42 ± 0.67	0.8995 ± 0.0760
AA loaded NE	12.40 ± 0.35	0.9946 ± 0.0033

## Data Availability

The dataset is available on request from the authors.

## Supplementary Materials

The following supporting information can be downloaded at https://www.mdpi.com/article/10.3390/nano16130793/s1, Supplemental Figure S1. Performance metrics for the evaluated models. A & B: Logistic regression, C & D: Decision trees, E & F: SVM. Supplemental Figure S2. A & B: Random Forest, C & D: Histogram Gradient Boosting, E & F: Extremely Random Forest.

## Abbreviations

The following abbreviations are used in this manuscript:

6SA	Anacardic 6-pentadecyl salicylic acid
NE	Nanoemulsion
ML	Machine Learning
SNEDDS	Self-Nanoemulsifying Drug Delivery System
AA	Anacardic acid
CQAs	Critical Quality Attributes
MPS	Mean particle size
PDI	Polydispersity index
HLB	Hydrophilic-lipophilic balance
TPSA	Total polar surface area
SVM	Support vector machine
ROC	Receiver operating characteristic
