# Supplementary material for: AI-Driven Design and Comparative Evaluation of SNEDDS for the Optimized Nanoencapsulation of Phytoextracts
Source: Nanomaterials (Basel). 2026 Jun 26;16(13):793. doi: 10.3390/nano16130793 (PMC13363240; doi:10.3390/nano16130793)

Supplemental Figure S1. Performance metrics for the evaluated models. A & B: Logistic regression, C & D: Decision trees, E & F: SVM.

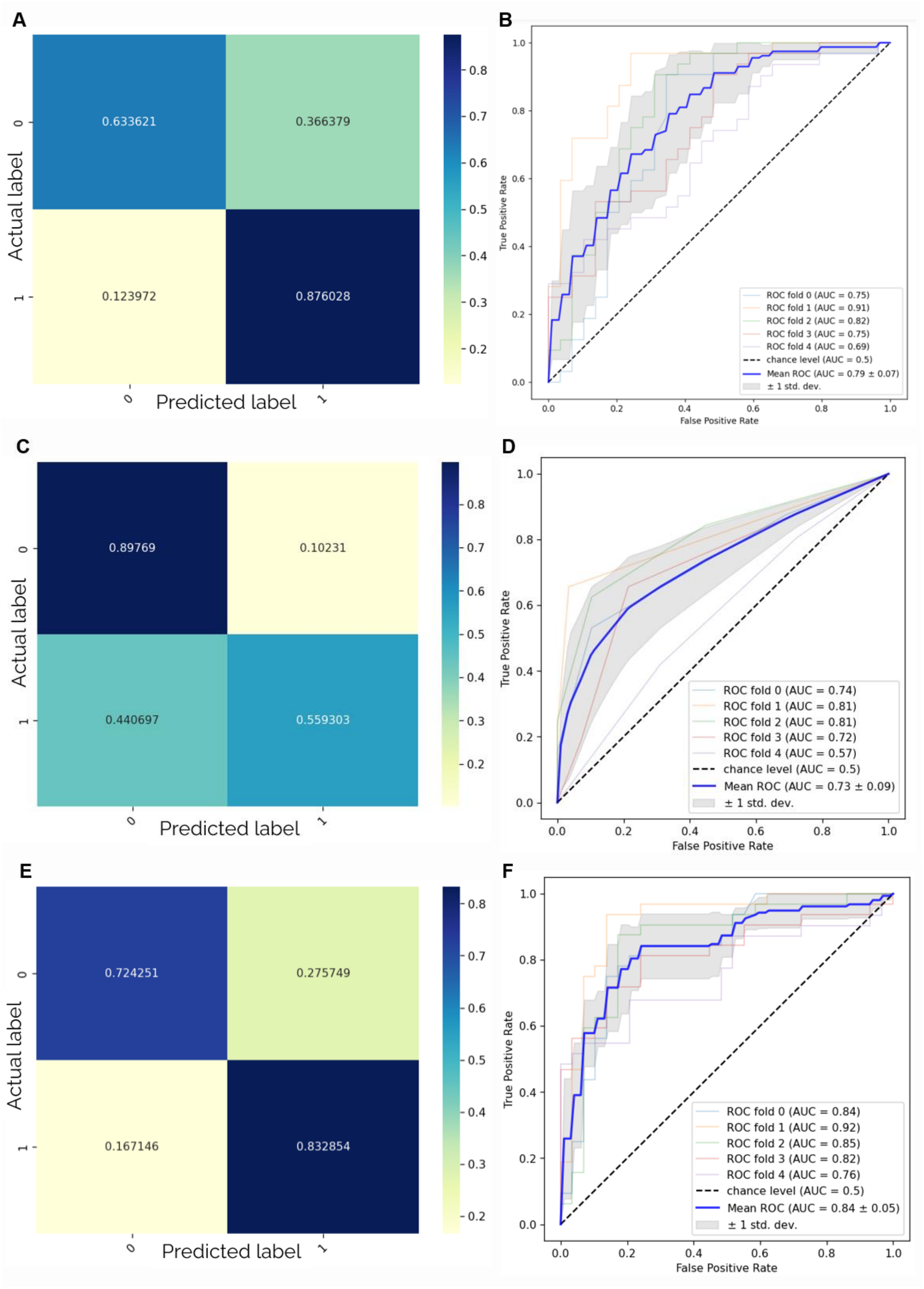

Supplemental Figure S2. A & B: Random Forest, C & D: Histogram Gradient Boosting, E & F: Extremely Random Forest.

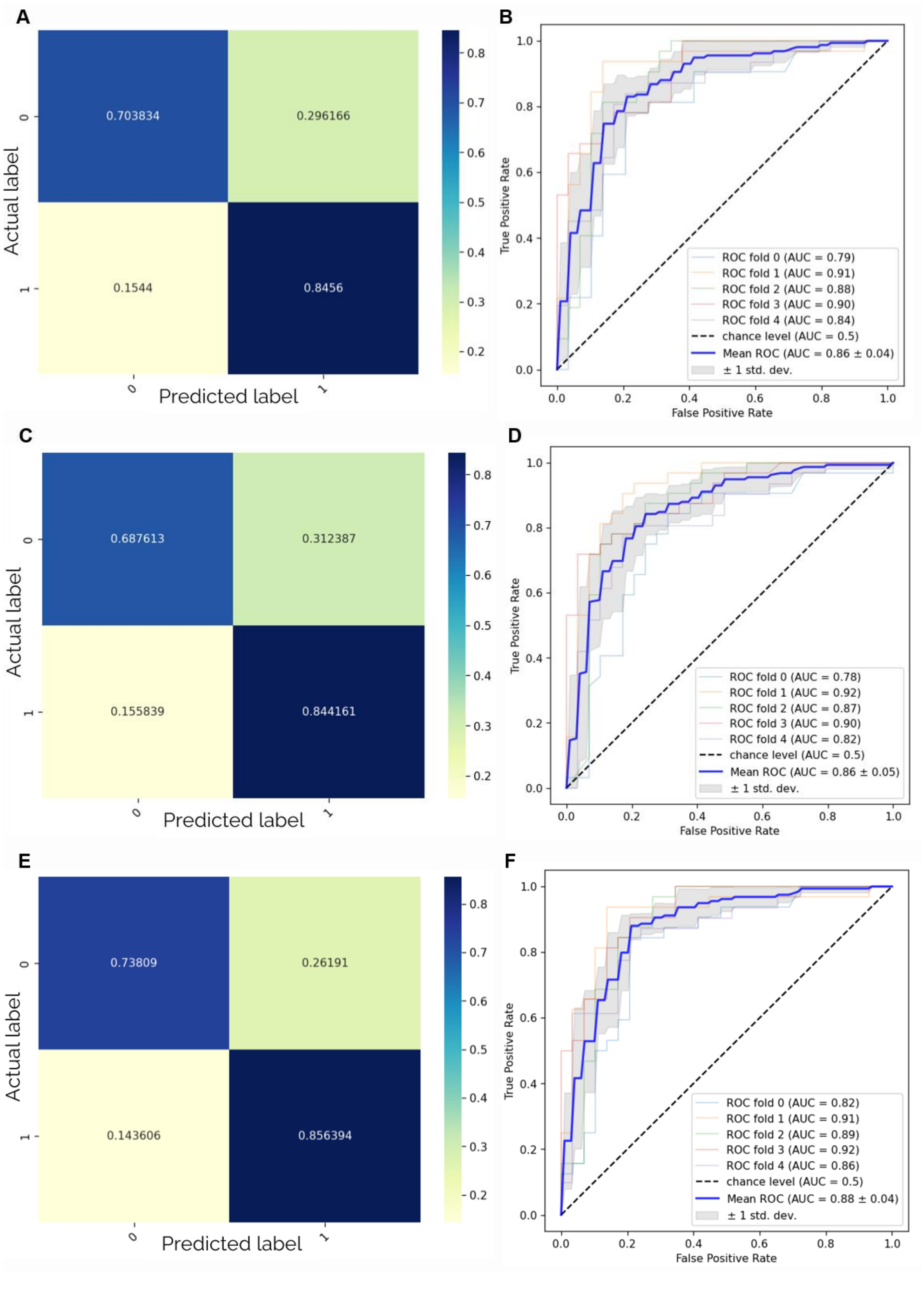

Supplement: Supplementary file 1 [file nanomaterials-16-00793-s001.zip › nanomaterials-4323423-supplementary.pdf]
